# Pennsylvania Hospital for the Insane

**Published:** 1846-12

**Authors:** 


*Pennsylvania Hospital for the Insane.*—We have received, (but deferred by accident to notice,) the report of Dr. Thos. S. Kirkbride, physician to the Pennsylvania Hospital for the Insane, during the year 1845.

The report shows that there has been admitted into the hospital, during the year for which the report was made, 767 patients, of whom 447 were males, the remaining 322 of the whole number were females. During the year, there had been nine deaths among the male, and eleven among the female patients. Eleven of these deaths were in patients who were admitted for incurable forms of disease. This institution is a private charitable institution, receiving no assistance from the city or state in which it is located, and expends all of its income for the benevolent objects of its foundation. From its foundation in 1752, up to 1841, there have been admitted 4366 insane patients to the benefits of this institution. It is proper that we should say, in this connection, that, during its early establishment, it received not only insane, but other sick patients.

This report sets forth, in addition to the operations and workings of the institution, an amount of statistical information, not only important as comprising a part of the report, but valuable for physiological reference.

Amongst the many philanthropic institutions of our country, few, if any, have greater claims upon the sympathies, or deserve more thanks for the benefits dispensed, than those institutions so widely distributed through the land, for the benefitting of that unfortunate class of the community which the founders of the one now under consideration had in view.

In closing this imperfect notice, in the quoted language of the report, we would say, "May the God of mercies bless the undertaking."

